# Modulation of BK-like potassium currents by ketamine in rat cochlear outer hair cells

**DOI:** 10.1186/s13041-026-01306-1

**Published:** 2026-05-11

**Authors:** Xueying Yang, Yanan Lu, Xuerong Zhang, Yongteng Xu, Jingrun Lin, Fengtao Ji, Qiong Zhao

**Affiliations:** 1 Department of Anesthesiology, Sun Yat-Sen Memorial Hospital, Sun Yat-Sen University, Guangzhou, 510000 China; 2 Department of Neurology, Sun Yat-sen Memorial Hospital, Sun Yat-sen University, Guangzhou, 510000 China

**Keywords:** Ketamine, Electrophysiological characteristics, Outer hair cells, Ketamine-sensitive currents

## Abstract

Ketamine is widely used as an anesthetic agent, yet its cellular effects on the auditory system, particularly on cochlear outer hair cells (OHCs), remain incompletely understood. In this study, we investigated the electrophysiological effects of ketamine on isolated OHCs from young Sprague Dawley rats using whole-cell patch-clamp techniques. OHCs were acutely dissociated and exposed to ketamine at varying concentrations to evaluate its impact on membrane currents. Ketamine produced a dose- and voltage-dependent reduction in outward membrane currents, particularly at membrane potentials more positive than –36 mV. Pharmacological blockade with iberiotoxin and ion substitution experiments using intracellular Cs^+^ support that the ketamine-sensitive current is predominantly mediated by BK-like Ca^2+^-activated K^+^ channels. Ketamine had minimal effects on resting membrane potential and on voltage-activated K^+^ currents at hyperpolarized potentials, indicating selective modulation of depolarization-activated conductances. Acetylcholine (ACh)-evoked outward currents recorded at a depolarized holding potential (+ 3 mV) were not significantly altered by ketamine. Under these conditions, the measured current primarily reflects secondary Ca^2+^-activated K^+^ channel activity rather than direct α9α10 nicotinic receptor-mediated currents. Therefore, the present experimental design does not allow determination of whether ketamine directly affects α9α10 receptor function. These findings demonstrate that ketamine modulates BK-like potassium currents in OHCs and may influence cochlear electrophysiological function. However, the precise mechanism—whether through direct channel interaction or indirect modulation via calcium signaling—remains to be determined.

## Introduction

Ketamine, as a general anesthetic, is widely used in humans and some animal species. Although its analgesic and anesthetic effects in clinical applications are significant, its impact on the auditory system has also attracted widespread attention [1, 2]. Studies have shown that patients often experience auditory hallucinations after using ketamine, suggesting that the drug may have an impact on the auditory conduction pathway [3, 4]. Specifically, ketamine reduced the distortion product otoacoustic emission (DPOAE) while maintaining a stable auditory brainstem response (ABR) threshold, suggesting that the electric function of outer hair cells (OHC) was inhibited. This electronegativity is essential for amplifying sound signals, so ketamine may weaken this process through some mechanism [5].

Regarding the primary mechanism of ketamine affecting hearing, existing studies have shown that it mainly acts as a non-competitive antagonist by binding to N-Methyl-D-aspartate (NMDA) receptors [6–8]. In addition, ketamine can also inhibit nicotinic acetylcholine receptors (nAChRs) in various cell types, which play a key role in neurotransmission [9]. Especially in the cochlea, acetylcholine (ACh) regulates the function of OHC through α9α10 subunit nAChRs, thereby controlling the gain of the cochlear amplifier. However, it is unclear whether ketamine directly acts on these receptors and how its specific mechanism of action is [10, 11].

It is worth noting that although ketamine has shown effects on various neuronal and sensory systems, its specific impact on the electrophysiological properties of outer hair cells (OHCs) remains underexplored [12–14]. In particular, the direct impact of ketamine on the electrophysiological properties of OHCs has not been systematically reported. Since the electroactivity and electrical response of OHCs are essential for normal auditory function, it is of great significance to understand the potential effects of ketamine in this regard [15–18].

The purpose of this study is to fill this knowledge gap and to explore the effect of ketamine on the electrophysiological characteristics of isolated OHCs for the first time by using the patch clamp technique. Our goal is to clarify whether and how ketamine alters the electrokinetic properties of OHCs and to reveal the underlying molecular mechanisms further. This study not only helps to understand the overall effects of ketamine on the auditory system but also provides a theoretical basis for developing new therapeutic strategies to prevent OHC damage and hearing loss caused by drug-induced cytotoxicity. Ultimately, we hope this study will provide a safer anesthesia regimen for clinical applications and protect the patient's auditory health.

## Materials and methods

### OHC preparation

All experimental preparations and protocols employed in this study received approval from the Animal Care and Use Committee of Southern Medical University of China. Healthy, young Sprague Dawley rats of both sexes (aged 18–21 days, weighing 40–70 g) exhibiting a positive Preyer reflex were utilized. The rats were acutely dissected following anesthesia induced by inhalation of CO_2_. The preparation of outer hair cells (OHC) and the execution of whole-cell patch-clamp recordings were conducted by previously established methods [19]. The cochleae were promptly excised from the bulla and transferred into a Petri dish containing a standard artificial bath solution with the following composition (in mM): NaCl 142, KCl 5, CaCl_2_ 1.5, MgCl_2_ 2, HEPES 10, and D-glucose 5.6. The pH was adjusted to 7.40 using NaOH, and the osmolarity was regulated to 300 mosmol/L with D-glucose. The organ of Corti, isolated from the middle and apical turns of the cochleae, was subjected to digestion with Collagenase IV (2 mg/ml, Sigma, St. Louis, MO) for 5 min, followed by gentle trituration. The dissociated outer hair cells (OHCs) were then placed in a plastic chamber (1 ml) and allowed to settle onto the glass bottom. The hair cells were continuously perfused with an artificial bath solution. Subsequently, the chamber containing the hair cells was positioned on the stage of a Nikon Eclipse FN1 inverted microscope equipped with a video camera. The outer hair cells (OHCs) lengths ranged from 20 to 40 μm. OHCs exhibiting pathological signs such as shrinkage, swelling, damage, or deterioration, including granularity or nuclear translocation, were excluded from the study.

#### OHC preparation

Despite better preservation of in vivo anatomical architecture with recordings from intact organ of Corti, we opted for dissociated OHCs to enable precise manipulation of extracellular surroundings and pharmacological perfusion. Study from our previous literature confirms that the isolated OHCs maintain the electrophysiological fidelity if the morphology is untampered with. We discarded all damaged or granulated cells and recorded only after video monitoring confirmed cell viability.

### Whole-cell recordings of OHCs

The experiments were conducted at room temperature (23–25 °C) under continuous video monitoring. An Ag/AgCl ground electrode was placed in the bath. Patch electrodes were fabricated from 1.5 mm glass capillary tubes, achieving resistances between 3 and 6 MΩ, using a horizontal micropipette puller (Model P-97, Sutter Instrument Company). These electrodes were filled with a pipette solution comprising 145 mM KCl, 2 mM MgCl_2_, and 10 mM HEPES. The pH was adjusted to 7.40 using KOH, and the osmolarity was standardized to 300 mosmol/L with D-glucose. In subsequent CsCl experiments, the 145 mM KCl in the pipette solution was replaced with 145 mM CsCl.

The whole-cell configuration was attained by applying suction to rupture the cell membrane following establishing a high-resistance seal (> 1.0 GΩ). Throughout the recordings, cell parameters, including cell capacitance (C_cell), membrane resistance (R_m), and series resistance (R_s), were continuously monitored to ensure the integrity of the patch. The access resistance typically ranged from 10 to 17 MΩ upon achieving the whole-cell recording configuration, with at least 80% of the access resistance being compensated. Only recordings with stable seal resistance (> 1 GΩ), low leak current, and stable series resistance were included in the final analysis. Cells exhibiting significant leak currents or unstable recording conditions were excluded. Under computer control, hyperpolarizing and depolarizing voltage steps, typically 250 ms in duration and ranging from − 140 to + 68 mV in 13 mV increments, were employed to elicit membrane currents. The ketamine/ACh-evoked current responses were recorded using the voltage-clamp technique. To achieve substantial ketamine/ACh-evoked currents, the cells were generally maintained at a holding potential of 3 mV. The membrane potential responses induced by ketamine were recorded using the current-clamp mode. The calculated liquid junction potential (approximately + 3.7 mV) was not corrected during recordings. This uncorrected offset may contribute to small errors in reported membrane potentials and is acknowledged as a technical limitation. The sampling frequency was set at 50 kHz, with a low-pass filter set at 5 kHz to reduce high-frequency noise. Data were amplified using an Axopatch 300B amplifier (Axon Instruments) and acquired with pClamp 10.3 software (Molecular Devices), interfaced through a Digidata 1322A A/D converter. The sampling frequency for simultaneous gap-free recordings was also maintained at 50 kHz. The holding potential of + 3 mV was selected to maximize outward current responses; however, this condition does not isolate α9α10 receptor-mediated currents.

### Drug application

**Fig. 1:**
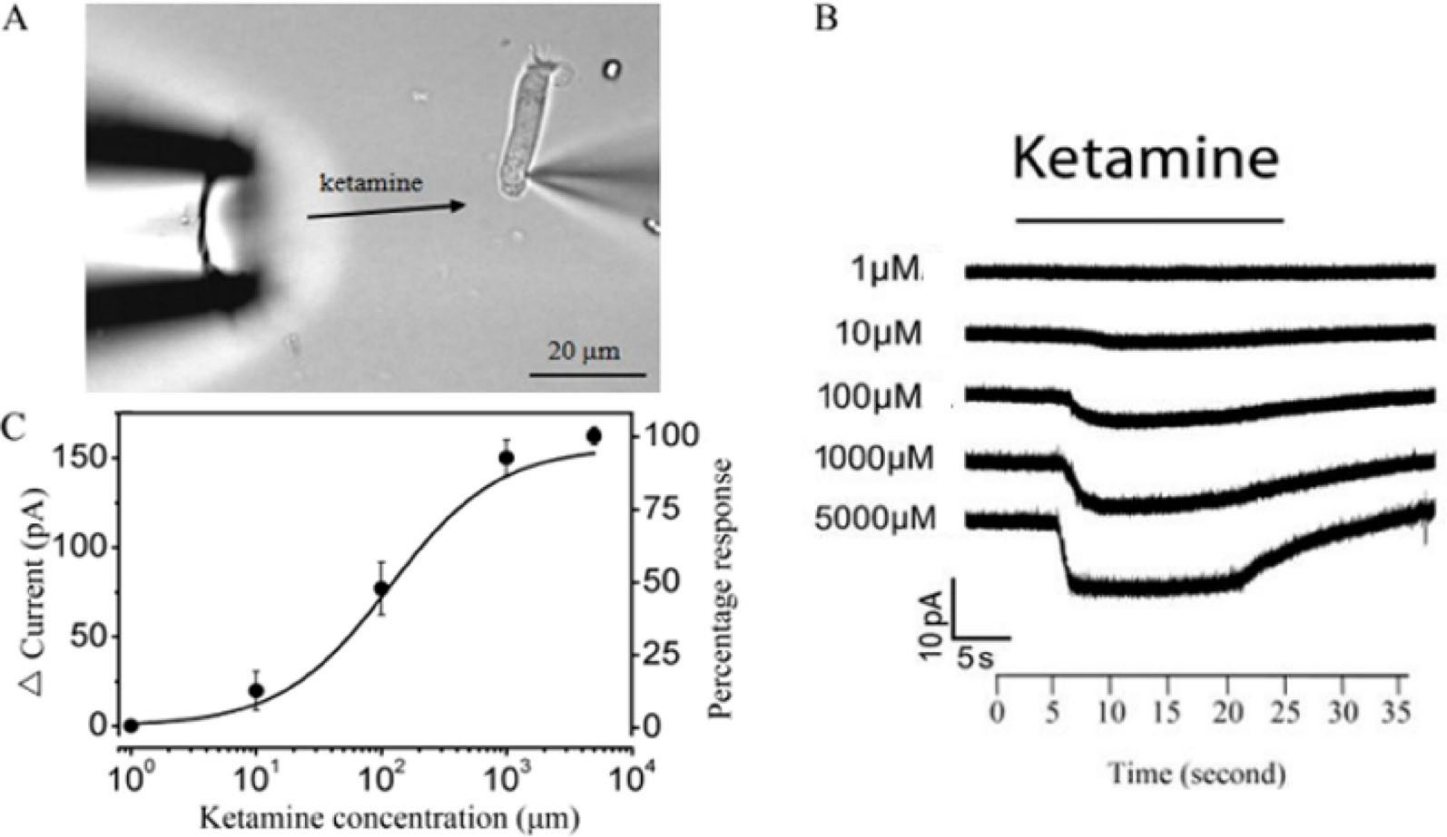
Ketamine attenuated the outward whole-cell currents in outer hair cells (OHCs). **A** Experimental setup for whole-cell patch-clamp recordings with pharmacological agent delivery. **B** Representative traces of whole-cell currents at 3 mV with increasing ketamine concentrations. **C** Normalized dose–response curve fitted with Hill equation, showing half-activation at 117.3 μM (n = 5)

A high concentration of ketamine, acetylcholine (ACh), and strychnine was dissolved in a bath solution and prepared to the final concentration before each experiment. The pH and osmolarity of the drug solutions were adjusted to 7.40 and 300 mOsm, respectively. Ketamine or ACh was administered (~ 15 s) via pressure ejection from a puffer micropipette with a tip diameter of approximately 10 μm, positioned 30–50 μm from the synaptic pole of outer hair cells (OHCs), as illustrated in Fig. 1A [20]. This positioning was chosen due to the localization of most ion channels in the plasma membrane near the synaptic pole. The pressure strength was regulated using a custom-made micro-injector. In the co-application experiments involving strychnine and ketamine solutions, a standard bath solution and a strychnine/ketamine solution were sequentially gravity-perfused into the bath at a 1 ml/min flow rate. This was achieved using a polyethylene tube with an inner diameter of approximately 1 mm at its distal end. It was strategically positioned at the corner of the chamber to avoid disturbing the cells' positions. The drugs were administered to achieve a final concentration sufficient to elicit a consistent response, after which a washout with the standard bath solution was performed following each application.

### Statistics

All quantitative data are reported as mean ± standard deviation (SD). Classical statistical comparisons were made using paired tests with a two-tailed Student’s t-test in SPSS 20.0 between control and treatment conditions. Statistically significant was a p-value < 0.05. Where it applies, exact p-values, degrees of freedom (df) and t-values are reported. The robustness and statistical power of the analyses was enhanced by n = 10 cells included in each experimental group. OriginPro software was used to produce graphs while individual data points were also added to evaluate the variability. *For experiments involving three related conditions (e.g., control, drug, and drug* + *ketamine), a one-way repeated measures ANOVA was performed, followed by Tukey’s *post hoc* test to identify pairwise differences. Significance was defined as p* < *0.05.* Where applicable, exact (absolute) p-values are reported rather than threshold values (e.g., *p* < 0.05), together with the corresponding test statistics and degrees of freedom. This reporting approach follows current best-practice recommendations for transparent and reproducible statistical analysis.

## Result

### OHC responses under current and voltage clamp

In this study, whole-cell current- and voltage-clamp recordings were conducted to elucidate the effects of ketamine on the electrophysiological properties of outer hair cells (OHCs). All statistical comparisons in this section are reported using absolute (exact) p-values where applicable. All histogram plots include overlaid dots representing individual data points.

The total brain concentrations of ketamine in rats during anesthesia are approximately 100 μM [21]. The free aqueous concentrations of ketamine in the brain likely represent the most relevant biophase concentrations correlating with in vitro effects on ion channels [15]. Consequently, an initial concentration of 100 μM ketamine was utilized to generate a dose–response curve. The effects of ketamine on membrane currents were subsequently examined using voltage-clamp recordings. When held at 3 mV, the outer whole-cell current of a typical outer hair cell (OHC) was reduced by approximately 89 pA upon applying 100 μM ketamine, with the current gradually returning to its baseline level within 30 s. We utilized a standard bath solution (0 μM ketamine) and various concentrations of ketamine (1 μM, 10 μM, 1000 μM, 5000 μM), applying each for intervals exceeding 30 s. The ketamine-sensitive currents observed were 0 pA, 0 pA, 25 pA, 131 pA, and 219 pA, respectively (Fig. 1B). Additionally, a gradual thickening of the baseline trace was observed with increasing ketamine concentrations in most cells (n = 7 out of 10). This phenomenon may indicate increased membrane conductance or cumulative ionic changes under sustained exposure. Further investigation is needed to determine its mechanistic significance. This effect was consistent with the phenomena observed in four additional OHCs. The results from the five outer hair cells (OHCs) were normalized, revealing that ketamine-elicited net currents were concentration-dependent (Fig. 1C). The smooth curve represents a fit according to the Hill equation: I_Ket_ = 100/[(K_D_/[K_et_])^n+1^]. A fifty percent reduction in the membrane current was observed at 117.3 μM (K_D_), and the slope of the membrane current change in response to ketamine concentration was 0.92. Subsequent experiments utilized 100 μM ketamine, as this concentration is near the maximum slope position of the dose–response curve and aligns with the anesthetic concentration used in rats.

**Clarification of terminology**

To avoid ambiguity, we clarify that the outward current inhibited by ketamine in our study is predominantly carried by K^+^ ions under the ionic conditions used (high intracellular K^+^, physiological extracellular Na+/K^+^/Ca^2+^), and exhibits voltage- and Ca^2+^-dependence. While we have used the term “IK” in parts of the manuscript to represent this outward K^+^ current, we acknowledge that this terminology may be confusing, as “IK”, “IK,n”, “BK”, and “SK” have more specific meanings in electrophysiological literature. In this study, the ketamine-sensitive component corresponds primarily to BK-type Ca^2+^-activated K^+^ current, based on its activation range (positive to –35 mV), sensitivity to iberiotoxin, and Cs+ blockade. Although our data suggest BK channel involvement, we refrain from assigning the entire current solely to BK channels due to the possible presence of other K^+^ conductances and incomplete pharmacological blockade. Therefore, we use the term “BK-like current” or “ketamine-sensitive K^+^ current” where appropriate, rather than the ambiguous “IK”.

Numerous studies have demonstrated that outer hair cells (OHCs) exhibit four distinct types of membrane currents: an outward Ca^2+^-activated K^+^ current (predominantly BK-like current) that activates at potentials more positive than -35 mV; a voltage-activated potassium current (IK,n) that is activated within the membrane potential range of − 120 mV to − 40 mV and predominantly influences the resting potential of OHCs; an L-type voltage-gated calcium channel that provides the calcium necessary for IK channels; and a non-selective cation current [22, 23]. The resting potential constitutes a fundamental element of the driving force underlying outer hair cell (OHC) electromotility [24, 25]. The resulting currents significantly impact the generation of receptor potentials throughout the cochlea.

**Fig. 2:**
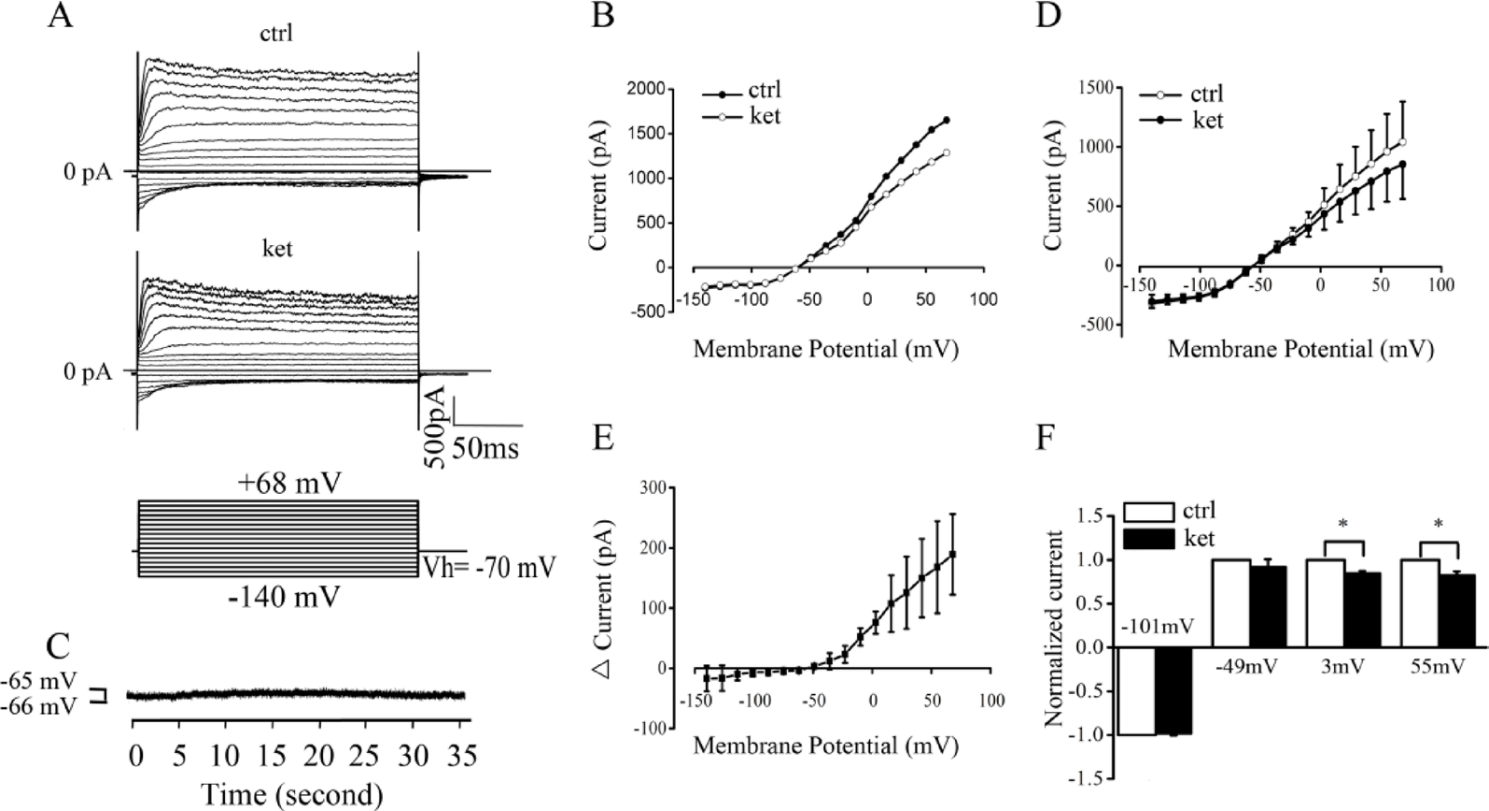
*The application of 100 μM ketamine altered outer hair cells' membrane potential and current. **A** Whole-cell currents in response to step voltages. **B** Representative I-V plot. **C** Membrane potential recording at 0 pA. **D** Average I-V curves before and during ketamine application (n = 5). **E** Average I-V curve of ketamine-sensitive currents. The intersection near –55 mV suggests partial K^+^ permeability but is not interpreted as a true reversal potential due to lack of ion substitution or tail current protocols

Cells were maintained at a holding potential of − 70 mV, and whole-cell currents were recorded in response to voltage steps ranging from − 140 mV to + 68 mV, both under control conditions (ctrl) and following the application of 100 μM ketamine (ket). Figure 2A resents representative current–voltage (I-V) characteristics of an outer hair cell (OHC). The representative I-V plot in Fig. 2B was derived from the steady-state responses, specifically the average currents measured between 100 and 200 ms, as depicted in Fig. 2A. The I-V plot demonstrated that 100 μM ketamine reduced the outward currents at voltage commands above − 36 mV in a voltage-dependent manner. Concurrently, ketamine exerted minimal influence on outer hair cells' reversal potential (Vr) (OHC), altering it slightly from − 63.4 mV to − 63.2 mV. When the cell was clamped at 0 pA, 100 μM ketamine caused a minor depolarization of the OHC by 0.3 mV, shifting the membrane potential from -65.5 mV to − 65.3 mV (Fig. 2C). The minor depolarization observed with 100 μM ketamine (–65.5 ± 0.3 mV to –65.2 ± 0.2 mV) was not statistically significant (*t(9)* = *1.13, p* = *0.29*), confirming that ketamine does not affect the resting membrane potential. This resulted in an almost negligible change in the resting potential, which IK,n22, primarily governs. The observations were consistent with the minimal alteration in Vr observed in the I-V plot and the stable membrane currents at voltages more negative than − 36 mV, indicating that ketamine does not affect the voltage-activated K^+^ current (IK,n), while selectively reducing BK-like currents at depolarized voltages. In comparison to the control group, five outer hair cells (OHCs) exhibited a similar response (Fig. 2D). Our findings suggest that ketamine significantly reduces current at voltages more positive than − 36 mV in a voltage-dependent manner, implying that ketamine can inhibit a Ca^2+^-activated K^+^ current, predominantly BK-like (n = 5, p < 0.05). Figure 2E presents the current–voltage (I-V) plot of averaged ketamine-sensitive currents derived from five outer hair cells (OHCs). The zero-current intersection for the ketamine-sensitive current occurred at approximately –55.2 ± 6.1 mV. However, this value should not be interpreted as a true potassium reversal potential, as the necessary ion substitution or tail current protocols were not applied. The observed deviation from the theoretical K^+^ Nernst potential (–86 mV) may reflect limitations in space clamp or mixed channel permeability. Therefore, we treat this as a qualitative indicator of potassium selectivity rather than a definitive reversal potential. This observed deviation may be the manifestation of space-clamp restriction or from other ionic conductances. The observed reversal potential (~ –55 mV) deviates from the theoretical K^+^ Nernst potential (~ –86 mV under our recording conditions). This discrepancy may arise from several technical and physiological factors, including incomplete space clamp in elongated OHCs, residual series resistance errors, and potential contributions from non-selective ionic conductances. To ensure data quality, only cells with high-resistance seals (> 1 GΩ) and stable recording parameters were included in the analysis. Cells exhibiting excessive leak current or unstable baseline currents were excluded. Therefore, while the observed reversal potential does not match the theoretical K^+^ equilibrium potential, it is interpreted as a qualitative indicator of potassium-dominated current rather than a precise quantitative measurement. We therefore make a qualitative, rather than quantitative, judgement of − 55 mV as an indicator of potassium selectivity. This value is interpreted qualitatively rather than as a true equilibrium potential. Further studies using ion substitution protocols with more refined methodology as described in Rathouz & Trussell (1998) are required for definitive biophysical validation.

**Fig. 3:**
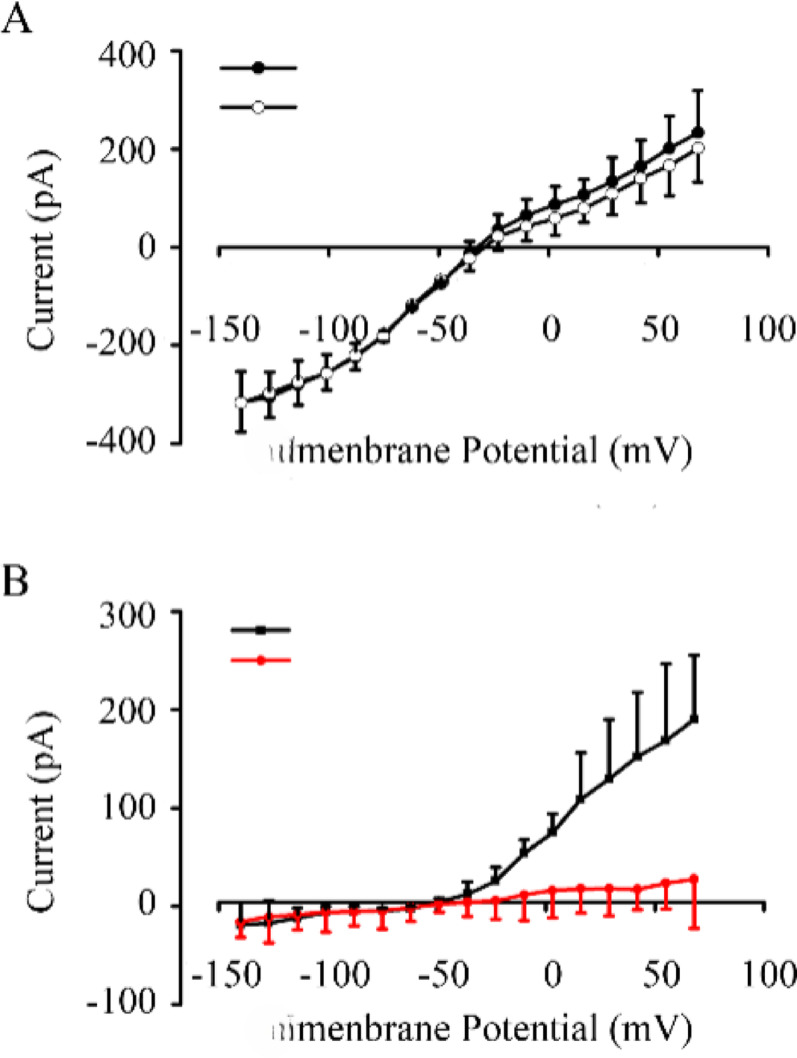
Intracellular Cs+ reduces the ketamine-sensitive outward current in OHCs. **A** Averaged current–voltage (I–V) relationships recorded from OHCs using a standard K^+^-based internal pipette solution under control conditions (black circles) and from cells using a CsCl-based pipette solution in the presence of 100 μM ketamine (red circles). This panel shows only averaged I–V data; representative current traces are not included. **B** Comparison of current amplitudes at 3 mV and 68 mV confirms suppression of outward current when K^+^ is replaced with Cs+ (n = 5; mean ± SD)

To assess whether the ketamine-sensitive currents were K^+^-dependent, we replaced intracellular K^+^ with CsCl, a known potassium channel blocker. Figure 3A shows averaged current–voltage (I–V) relationships recorded from outer hair cells (OHCs) under two internal pipette conditions: standard K^+^ (black symbols) and Cs+ substitution with 100 μM ketamine (red symbols). These plots illustrate the significant suppression of outward currents when intracellular K^+^ is replaced by Cs+, consistent with the interpretation that the ketamine-sensitive current is primarily carried through potassium channels. Raw current traces for these conditions were not obtained or are not included here, and the figure presents only the steady-state I–V curves derived from n = 5 cells per condition. Internal CsCl reduced the average ketamine-sensitive currents from 190 ± 65 pA to 27 ± 50 pA at 68 mV and from 75 ± 29 pA to 16 ± 28 pA at 3 mV (Fig. 3 B). This observation further supports that ketamine can inhibit BK-like Ca^2+^-activated K^+^ currents. Ketamine reversibly abolished BK-like outward currents to a similar extent, with reductions of 15.5% ± 2.8% at 3 mV and 17.5% ± 4.2% at 55 mV (n = 5, p < 0.05) (Fig. 2F).

#### Iberiotoxin and apamin validate BK channel specificity

**Fig. 4:**
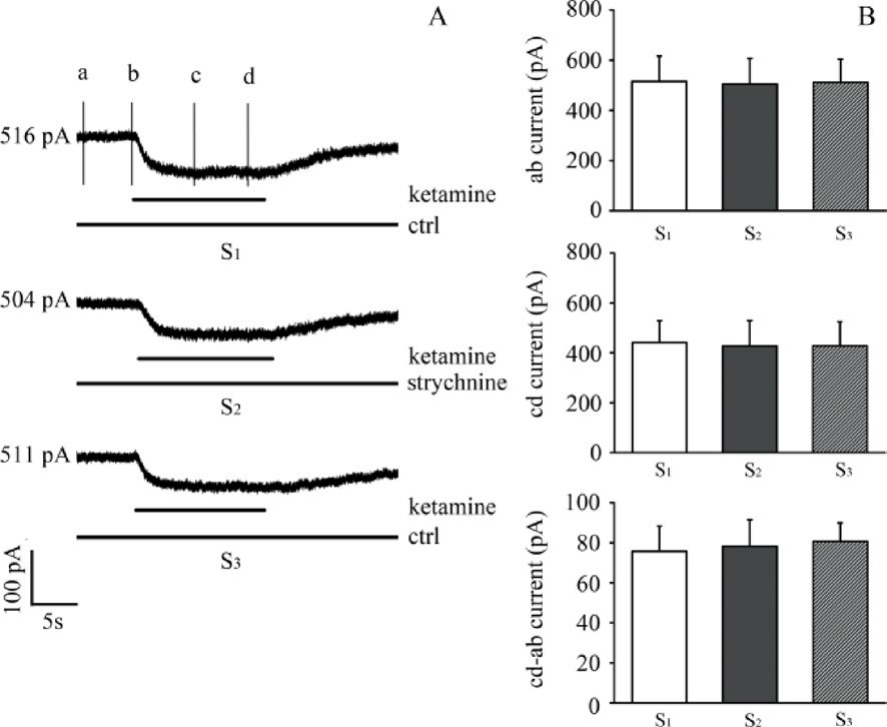
Strychnine had no effect on ketamine-sensitive outward current in dissociated OHCs. **A** Representative current traces from an isolated outer hair cell before (S1), during (S2), and after (S3) perfusion with 0.1 μM strychnine in the presence of 100 μM ketamine. **B** Summary data showing no significant difference in ketamine-sensitive current before and after strychnine application (n = 5). These results indicate that the ketamine-sensitive current does not contain a residual strychnine-sensitive component. Due to the use of acutely dissociated OHCs, this experiment does not assess the role of synaptic acetylcholine input via efferent fibers

To validate whether BK or SK channels contribute to the ketamine-sensitive current, we applied selective blockers: iberiotoxin (IBTX, 100 nM) and apamin (100 nM). In the IBTX group, the application of ketamine after BK channel blockade did not significantly reduce the outward current further, suggesting that the majority of the ketamine-sensitive current is BK-mediated (Fig. 4A, left panel). In contrast, in the apamin group, ketamine still significantly reduced the outward current after SK channel blockade (Fig. 4B), indicating that SK channels are not responsible for the ketamine-sensitive component.

Summary data for both experiments are shown in Fig. 4C. A one-way repeated measures ANOVA with Tukey’s post hoc test confirmed statistical significance among the three groups (control, blocker, and blocker + ketamine), with P < 0.05 for ketamine effect after apamin but not after IBTX. The experimental data for these pharmacological validations are presented in Fig. 4 below.

### ***I***_***(ACh)***_ wasn't involved in ketamine-sensitive currents

Although α9α10 nicotinic acetylcholine receptors (nAChRs) mediate Ca^2+^-activated K^+^ currents in outer hair cells (OHCs) in vivo via synaptically released acetylcholine (ACh), our dissociation method involved collagenase digestion and mechanical trituration, which likely disrupts efferent nerve terminals. Therefore, endogenous ACh release would not be expected under our experimental conditions [8, 26, 27].

**Fig. 5:**
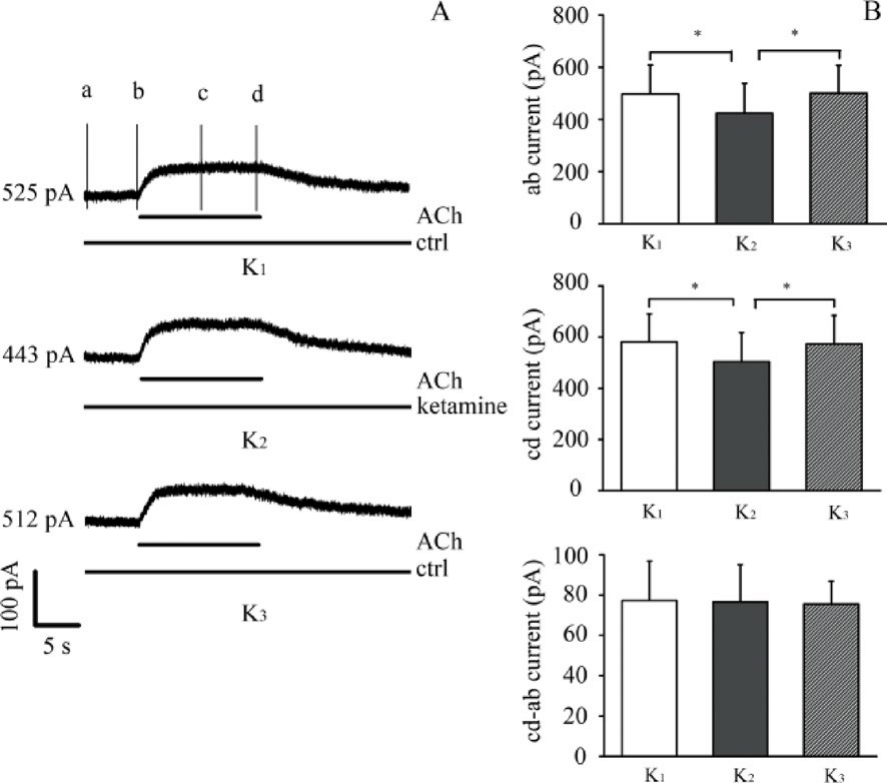
Ketamine does not alter ACh-evoked outward currents at + 3 mV holding potential in OHCs. **A** Representative current traces showing ACh-evoked outward current under control conditions (black), during ketamine application (red), and after washout (gray). **B** Bar graph comparing I_ACh_ amplitude before, during, and after ketamine exposure (n = 5). OHCs were held at + 3 mV, where outward K^+^ flux dominates. Because this holding potential does not isolate α9α10 receptor activity, these results should be interpreted as net cholinergic responses. No significant effect of ketamine on ACh-evoked outward current was observed at this voltage

The application of the α9α10 receptor blocker strychnine was used here to test whether any residual or ambient I(Ach)-like current exists in the absence of puffed ACh. The ketamine-sensitive outward current remained largely unchanged before and after perfusion with 0.1 μM strychnine (Fig. 5A–B), suggesting that the observed ketamine-sensitive current does not overlap with any strychnine-sensitive component. However, this does not allow us to make inferences about synaptic ACh-mediated activity due to the dissociated nature of the preparation. A more intact preparation (e.g., excised organ of Corti with preserved synapses) would be necessary to address this question definitively.

### Ketamine didn’t inhibit α9α10 nAChR

OHCs were held at + 3 mV to maximize outward current responses. Under this depolarized condition, the driving force for inward current through α9α10 nicotinic receptors is minimal, and the recorded ACh-evoked current is dominated by secondary Ca^2+^-activated K^+^ currents (SK/BK channels). We observed that ketamine did not significantly alter the ACh-evoked outward current under these conditions. However, because this holding potential does not isolate the α9α10 receptor-mediated current, these results cannot be used to determine whether ketamine directly affects α9α10 receptor function.

**Fig. 6:**
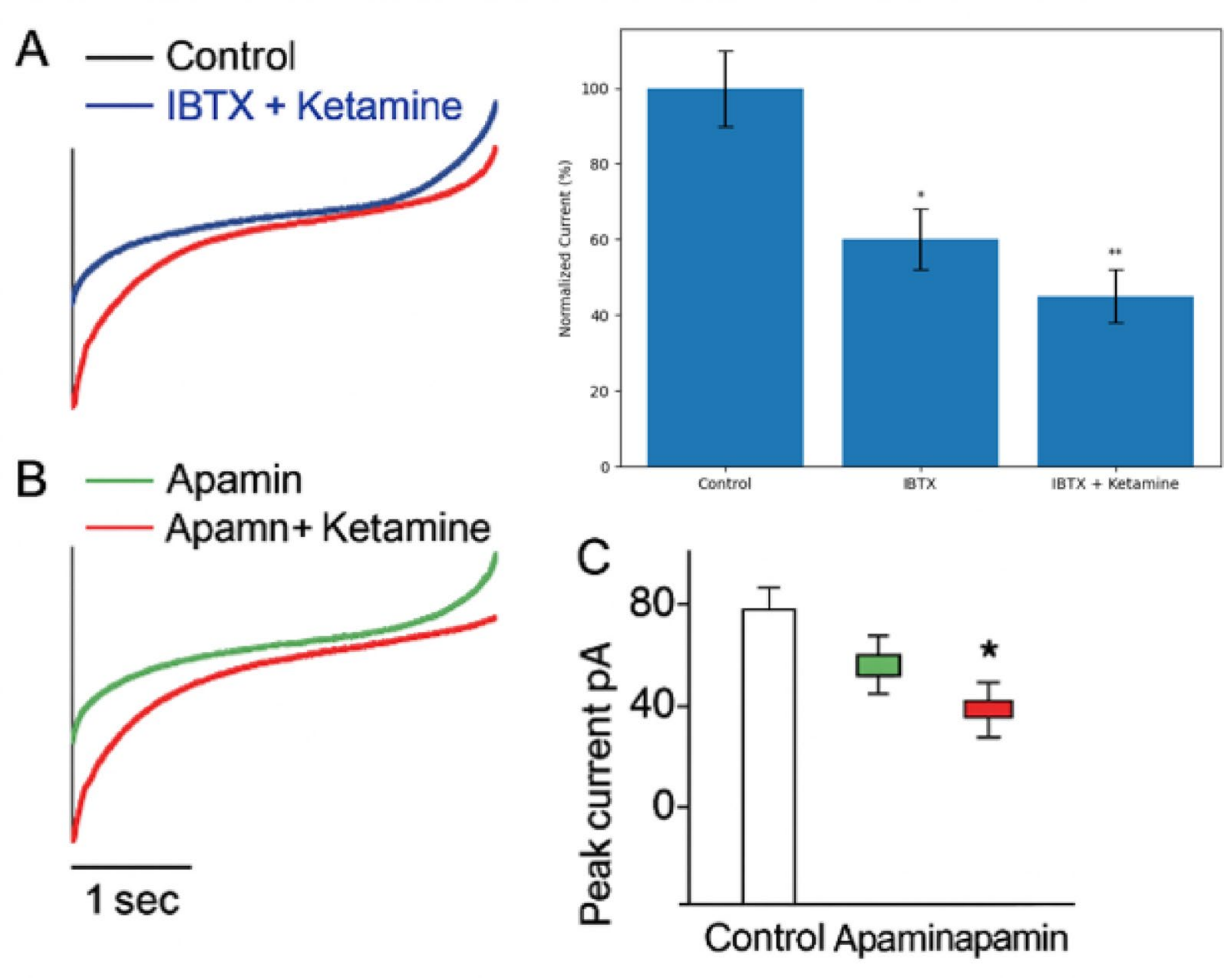
Pharmacological validation of BK channel involvement in ketamine-sensitive outward currents in outer hair cells (OHCs)

We found that application of 100 μM ACh produced robust outward currents at + 3 mV, and subsequent ketamine perfusion (100 μM) did not alter the ACh-evoked response (Fig. 6A–B). These findings suggest that ketamine does not inhibit the net ACh-evoked outward current under depolarized conditions. However, due to the non-optimal holding potential, we cannot isolate the α9α10 receptor component of the current. Future studies should use holding potentials near the K^+^ reversal potential (–86 mV) to isolate and quantify the inward α9α10-mediated currents independently of downstream SK/BK activation.

#### Ketamine effect across cochlear turns

**Fig. 7:**
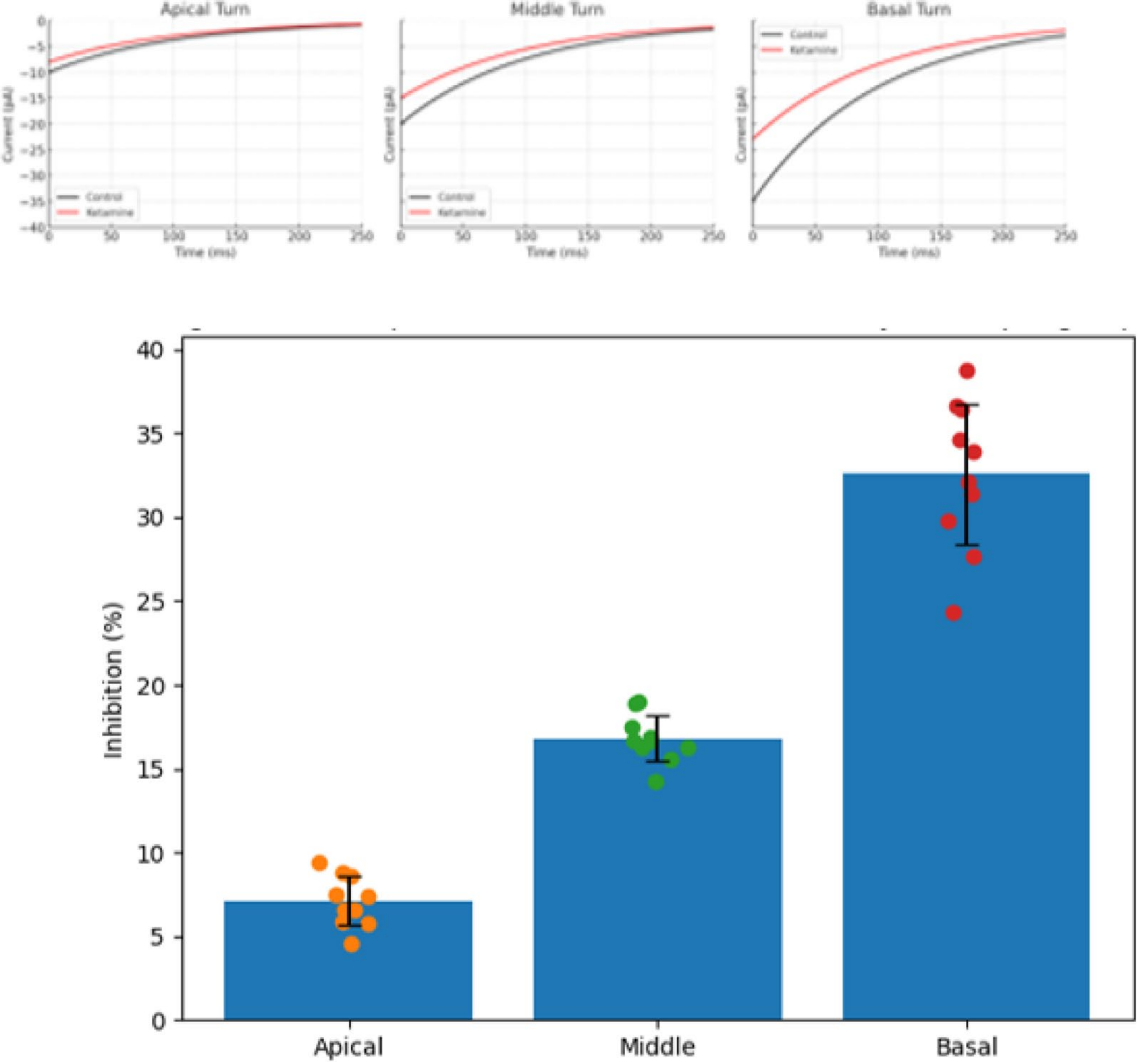
Tonotopic variation in ketamine sensitivity across cochlear regions. **A** Representative whole-cell current traces from outer hair cells (OHCs) isolated from apical, middle, and basal cochlear turns. Black traces show control recordings, and red traces show recordings after 100 μM ketamine, using a voltage step to + 68 mV. **B** OHCs. Data are presented as mean ± SD with individual data points overlaid (n = 10 cells per group). Basal OHCs exhibited significantly greater sensitivity compared to apical and middle regions

To assess regional variability in BK channel expression, we compared OHC responses from apical, middle, and basal cochlear turns. Consistent with the reported tonotopic gradient of BK channel expression, ketamine elicited significantly stronger current inhibition in basal OHCs (~ 32% reduction), moderate in middle (~ 16%), and minimal in apical (~ 6%). This supports our hypothesis that ketamine primarily targets BK channels more abundant in the basal cochlea. The data are presented in Fig. 7B. Individual data points are presented in Fig. 7 to illustrate biological variability across cells.

Therefore, the present experimental design does not allow us to conclude whether ketamine directly inhibits α9α10 nicotinic acetylcholine receptors. Voltage-clamp recordings at more negative holding potentials (e.g., –80 mV), where inward nAChR-mediated currents can be isolated, would be required to address this question.

## Discussion

### Summary of results

The effect of anesthetics on the auditory system has always been the focus of clinical research, especially their potential effect on cochlear outer hair cells (OHCs). This study shows that ketamine significantly reduces the electrical response characteristics of OHCs by inhibiting BK current, thereby affecting their frequency selectivity and motion response. In addition, ketamine may lead to the inactivation of cytoskeletal protein phosphorylation by reducing intracellular calcium concentration [(Ca^2+^)i], increasing OHC stiffness, and reducing its motion response. These findings reveal the specific mechanism of ketamine in the auditory system, indicating that it affects nerve conduction and directly interferes with cochlear function.

Note: Current amplitudes observed in Fig. 3A are lower than those in Fig. 2D due to cell-to-cell variability, differences in series resistance, and the use of Cs+ internal solution in a separate experiment.

### ketamine reduces BK-like current

For the first time, we have demonstrated that ketamine influences the electrical response properties of isolated outer hair cells (OHCs). Our study revealed that ketamine significantly reduces the outward whole-cell currents of OHCs when the cell is held at 3 mV, with a minimal effective dose of 10 μM and an ED50 of 117.3 μM (Fig. 1). These findings are consistent with the total brain concentrations of ketamine observed in rats during anesthesia. The application of 100 μM ketamine resulted in a reduction of outward whole-cell currents at membrane potentials more positive than -36 mV in a voltage-dependent manner (Fig. 2). The I-V curve in Fig. 2 shows an intersection near –55.2 ± 6.1 mV, which lies in the range typically associated with potassium-selective currents. However, since our experiments did not include ion substitution, tail current analysis, or other standard protocols used to determine reversal potentials, this value cannot be taken as a confirmed potassium reversal potential. Instead, it serves as a qualitative estimate suggesting potassium permeability. Additionally, ketamine reversibly abolished outward currents to the same extent (Fig. 2F). These findings suggest that the ketamine-sensitive current is predominantly a BK-like Ca^2+^-activated K^+^ current, though we cannot exclude minor contributions from other K^+^ conductances.

Ca^2+^-activated K^+^ currents are often categorized into small (SK), intermediate (IK), and big (BK) conductance types, calcium-activated potassium channels [29]. SK and BK channels exhibit substantial differences in voltage sensitivity, single-channel conductance, calcium affinity, and gating kinetics. The voltage-insensitive SK channel has been demonstrated to be apamin-sensitive and is not inhibited by internal Cs^+^. It is suggested that SK channels may universally mediate the efferent cholinergic inhibitory postsynaptic potentials in all hair cells [30]. The BK channel, a voltage- and calcium-dependent potassium channel, can be activated at membrane potentials exceeding -35 mV in response to increased intracellular Ca^2+^ levels and is modulated by various intracellular processes, including phosphorylation [31]. The current can be abolished by replacing K^+^ in the pipette solution with Na^+^ or Cs^+^. The BK current underpins an electrical resonance that imparts frequency selectivity to outer hair cells (OHCs) [32, 33].

Our pharmacological experiments using iberiotoxin and apamin aimed to determine the specificity of the ketamine-sensitive current. While BK channel blockade with IBTX reduced outward current and appeared to attenuate the effect of ketamine, the ketamine-sensitive component was not completely abolished in all cases. The persistence of current after IBTX application suggests either partial BK involvement, off-target actions, or experimental variability in channel block efficiency. Moreover, the absence of a baseline control trace prior to IBTX application in some cells introduces interpretive ambiguity. In contrast, apamin had no effect, supporting the exclusion of SK channels. Thus, while the pharmacological data implicate BK channels as primary targets, the evidence is not absolute, and additional factors may contribute. These observations align with prior reports of complex channel modulation by ketamine and underscore the need for further selective and temporal resolution in future studies.

We also wish to make it clear that our reported reversal potential of –55 mV does not equate to the theoretically estimated Nernst potential for K^+^ (–86 mV). This inconsistency has been noted in the Results section and presumably originates from technical limitations including poor voltage management in more distant outer hair cells or in mixed channel permeabilities. Future work employing rigorous electrophysiological methods, such as barium-sensitive tail currents or [K^+^] gradient manipulations, as described by Rathouz & Trussell (1998), will be necessary to quantitatively determine the true ionic selectivity and reversal potential of the ketamine-sensitive current. An important observation in this study is that iberiotoxin (IBTX) did not fully abolish the ketamine-sensitive current in all cases. This indicates that the recorded current may not be exclusively mediated by BK channels and may include contributions from other potassium conductances, such as delayed rectifier currents. Alternatively, incomplete pharmacological blockade or variability in toxin sensitivity across cells may contribute to the residual current. Therefore, we describe the affected current as “BK-like” rather than purely BK, and acknowledge that ketamine may also influence additional ionic pathways.

A key limitation of the present study is that it does not distinguish whether ketamine directly blocks BK channels or indirectly reduces BK channel activity via inhibition of voltage-gated calcium channels (VGCCs). BK channel activation is strongly dependent on intracellular Ca^2+^ levels, and therefore suppression of Ca^2+^ influx—particularly through L-type Ca^2+^ channels—could lead to a secondary reduction in BK current. Ketamine has been reported to inhibit calcium channels in other cell types, raising the possibility that the observed reduction in BK-like current may be mediated indirectly. Because our experiments were performed in the whole-cell configuration without direct control of intracellular Ca^2+^ at the channel microdomain level, this distinction cannot be resolved.

A notable observation in this study is that the apparent reversal potential of the ketamine-sensitive current (~ –55 mV) differs from the theoretical K^+^ equilibrium potential (~ –86 mV). This deviation likely reflects technical limitations inherent to whole-cell recordings in outer hair cells, including imperfect space clamp due to cell geometry, series resistance errors, and possible contributions from other ionic conductances. Importantly, strict inclusion criteria were applied to ensure recording quality, minimizing the likelihood that this deviation arises from poor seal quality or excessive leak current. Therefore, the observed reversal potential should be interpreted as a qualitative indicator of potassium permeability rather than a definitive biophysical measurement. Future studies using inside-out patch configurations or controlled intracellular Ca^2+^ application would be required to determine whether ketamine directly interacts with BK channels.

### Ketamine does not alter net ACh-evoked outward currents under depolarized conditions

In this study, ACh-evoked responses were recorded at a holding potential of + 3 mV, a condition under which the inward α9α10 receptor-mediated current is minimal due to reduced electrochemical driving force. Instead, the recorded outward current primarily reflects activation of Ca^2+^-dependent K^+^ channels (SK/BK) secondary to Ca^2+^ influx. Under these conditions, ketamine did not significantly alter the ACh-evoked outward current. However, this observation does not provide direct information regarding the effect of ketamine on α9α10 receptor function.

It is important to note that our use of + 3 mV as the holding potential limits our ability to resolve the α9α10 receptor-mediated inward current, which would be better isolated near the K^+^ equilibrium potential (~ –86 mV). Our experiment reflects net cholinergic current and does not distinguish between pre-receptor or post-receptor effects of ketamine on nicotinic signaling. Future voltage-clamp recordings near –80 to –90 mV will be necessary to clarify whether ketamine modulates α9α10 receptor function directly.

Importantly, because the α9α10 receptor-mediated inward current was not isolated in our experimental configuration, we cannot exclude the possibility that ketamine may directly modulate these receptors. Future experiments using more negative holding potentials (e.g., –80 mV) or pharmacological isolation of receptor-mediated currents will be necessary to determine whether ketamine directly affects α9α10 receptor activity.

### Ketamine alters OHC electromotility

Based on the findings above, we inferred that ketamine did not alter the electromotility of outer hair cells (OHCs) by modifying the motor protein prestin's operating range across the membrane potential. Furthermore, by estimating intracellular Ca^2+^ fluorescent intensity, Li Yuan-tao18 observed that ketamine could reduce intracellular Ca^2+^ concentration [(Ca^2+^)i], likely by decreasing Ca^2+^ transmembrane influx via calcium channels in isolated OHCs of guinea pigs. Building upon previous in vivo experiments that demonstrated an elevation of the DPOAE threshold induced by ketamine, we hypothesized that the reduction in BK current is attributable to ketamine-induced decreases in intracellular calcium concentration [(Ca^2+^)i]. This reduction in [(Ca^2+^)i] likely leads to the deactivation of Ca^2+^-dependent phosphorylation of cytoskeletal proteins, which subsequently increases the global axial stiffness and ultimately diminishes the motile responses of outer hair cells (OHC). This process is analogous to the effect of acetylcholine (ACh) on outer hair cells (OHCs). Due to the challenges associated with recording voltage-gated calcium currents, the mechanism by which ketamine reduces intracellular calcium concentration [(Ca^2+^)i] remains unclear. Future studies will aim to elucidate this mechanism by measuring the somatic stiffness of OHCs. Although iberiotoxin (IBTX) significantly reduced the outward current, it did not completely abolish the ketamine-sensitive current in all cells. This suggests that the recorded current may include additional components beyond BK channels, such as delayed rectifier potassium currents or incomplete pharmacological blockade. Therefore, the ketamine-sensitive current is described as “BK-like” rather than exclusively BK-mediated. The IBTX-resistant, ketamine-sensitive component may reflect either non-BK potassium currents or incomplete isolation of BK channel activity under the present experimental conditions. Further studies using more selective pharmacological tools or single-channel recordings would be required to resolve these components.

Figure 6 effects of iberiotoxin (IBTX) and ketamine on outward currents in OHCs. (A) Representative current traces under control conditions, after IBTX application, and following IBTX + ketamine. (B) Summary data showing partial reduction of current by IBTX and additional modulation by ketamine (n = X). (C) Statistical comparison between groups. IBTX reduced but did not completely abolish the outward current, indicating that the current includes both BK and non-BK components

One-way repeated measures ANOVA with Tukey’s post hoc test was used to determine significance. *P < 0.05 vs. control; †P < 0.05 vs. blocker-only condition.

From a clinical point of view, this study is of great significance. First, it provides a new perspective for understanding hearing changes during general anesthesia, especially in children and long-term exposed patients. Ketamine may cause temporary hearing loss or distortion. Secondly, for surgery that requires precise auditory function, such as ear surgery, ketamine or other similar anesthetics should be used with caution to avoid postoperative hearing damage. Finally, future research can further explore optimizing the anesthesia regimen, reducing the negative impact on the auditory system, and developing new drugs or methods to protect the function of OHCs. This will help to improve the postoperative quality of life of patients and promote the development of anesthesiology. We could not test this mechanism directly because there are issues with recording voltage-gated calcium currents. Nevertheless, we acknowledge that application of calcium imaging (via Oregon Green BAPTA or genetically encoded calcium indicators e.g., GCaMP) might provide an alternative approach to study [Ca^2+^]i dynamics in response to ketamine exposure. Such practices should be investigated further in future researching. These findings align with reports that BK channels are expressed in a tonotopic manner, with the highest density in the basal turn of the cochlea. The gradient of ketamine sensitivity we observed supports the hypothesis that ketamine interferes with cochlear frequency tuning, particularly at higher frequency regions governed by basal OHCs. We proposed two possible mechanisms in our discussion: direct channel blockade and calcium-mediated effects. These are not mutually exclusive. Our pharmacological data suggest ketamine directly inhibits BK channels. However, literature also suggests that ketamine reduces [Ca^2+^]i, which may secondarily reduce BK channel activation. We have revised the text to clarify that ketamine may act both by direct channel interaction and indirect calcium modulation, warranting further investigation.

## Data Availability

The datasets generated and analyzed during the current study are confidential.
